# Central fever in patients with spontaneous intracerebral hemorrhage: predicting factors and impact on outcome

**DOI:** 10.1186/s12883-015-0258-8

**Published:** 2015-02-04

**Authors:** Asaf Honig, Samer Michael, Ruth Eliahou, Ronen R Leker

**Affiliations:** Departments of Neurology, the Agnes Ginges Center of Neurogenetics, Hebrew University-Hadassah Medical Center, P.O. Box 12000, Jerusalem, 91120 Israel; Departments of Radiology, Hadassah-Hebrew University Medical Center, Jerusalem, Israel

**Keywords:** Stroke, Cerebrovascular disease, Intracerebral hemorrhage, Fever

## Abstract

**Background:**

Central fever (CF) is defined as elevated temperature with no identifiable cause. We aimed to identify risk factors for developing CF among patients with spontaneous intracerebral hemorrhage (ICH) and to evaluate the impact of CF on outcome.

**Methods:**

Patients included in our prospective stroke registry between 1/1/09 and 1/10/10 were studied. We identified patients with CF as those with a temperature ≥38.3°C without evidence for infection or drug fever. Patients with CF were compared to those without fever and those with infectious fever. Demographics, risk factors and imaging data as well as outcome parameters were reviewed.

**Results:**

We identified 95 patients with spontaneous ICH (median age 76, median admission NIHSS 9). CF was identified in 30 patients (32%), infectious etiology was found in 9 patients (9%) and the remaining patients did not develop fever. Baseline variables were similar between the groups except for intra-ventricular extension of the ICH (IVH) and larger ICH volumes that were more common in the CF group (OR = 4.667, 95% CI 1.658-13.135 and OR = 1.013/ml, 95% CI 1.004-1.021). Outcome analysis showed higher mortality rates (80% vs. 36%, p < 0.001) and lower rates of favorable functional outcome defined as a modified Rankin score ≤ 2 at 90 days (0% vs. 53%, p < 0.001) in the CF group.

**Conclusions:**

The risk of CF is increased in patients with larger ICH and in those with IVH. CF negatively impacts outcome in patients with ICH.

## Background

Intra-cerebral hemorrhage (ICH) is the most common non-ischemic cause of stroke [1,2] and is associated with very high morbidity and mortality rates [3-5].

The low survival rates of patients who suffer from ICH emphasize the importance of identifying prognostic factors. Previous research suggests that prognosis after ICH depends on hematoma location and size, neurological disability and level of consciousness at presentation, age, comorbidities, preceding anti-coagulant therapy and hyperthermia [3,4,6-10].

Hyperthermia was found to have deleterious effects on outcome in experimental models of brain injury [11-13]. Similar deleterious effects on outcome were found in patients with either ischemic or hemorrhagic stroke [14-18]. Moreover, brain temperature elevations have been associated with elevated intracranial pressure after subarachnoid hemorrhage (SAH) and traumatic brain injury (TBI) in the absence of infection [19].

Central fever (CF) was initially described as a rapid increase in core body temperature and icy cold extremities in the immediate aftermath of brain surgery [20]. Commichau et al. [21] found that fever (defined as temperature > 38.3°C) was observed in 23% of neurological intensive care unit (NICU) patients. Among them, 42% were associated with an identified infection but in 28% no explanation for the increased temperature could be identified suggesting a central origin of the fever. It is hypothesized that damage to any of the structures involved in temperature homeostasis pathways including coetaneous thermal receptors, spinal cord, midbrain and hypothalamus may cause CF [22-24]. Indeed, several studies have shown correlations between lesion size, type and location and the development of fever after brain injury [25-27].

The current study attempted to examine whether CF is associated with spontaneous ICH, whether factors predicting the development of CF can be identified and whether CF influences outcome in patients with ICH.

## Methods

We prospectively recruited consecutive patients presenting with ICH into our stroke registry. The institutional review board (Hadassah Medical Organization) authorized anonymous inclusion of patients into the consecutive data base without getting informed consent (approval # HMO-09-0277).

In the current analysis we included patients with ICH admitted between 1.1.2009 and 1.10.2010. The diagnosis of ICH was established according to clinical findings and a baseline non-contrast CT scan that showed the hemorrhage.

We studied demographics and cerebrovascular risk profile. Patients had a follow up non-contrast CT at 24 hours from treatment. According to this follow up CT, location and volume of hematoma, presence of subarachnoid or intraventricular hemorrhage were accrued. The volume of the hematoma was assessed with the ABC/2 formula [28]. In every case of atypical presentation of ICH further radiologic evaluation using CT angiography, angiography and MR imaging were performed. Every case of ICH that resulted from an etiology other than spontaneous ICH was excluded from the study. Another measure taken to ascertain this was a follow up MRI performed on 16 patients after full hematoma resolution and failed to reveal any underlying structural etiology.

Neurological severity at presentation was measured with the National Institutes of Health Stroke Scale (NIHSS) [26].

All patients were treated in an intensive care unit for at least 24 hours. Temperature data was collected for the first week after admission. Only patients with full data sets of temperature during the first week of admission were included in the study. Body temperature was measured at least three times daily. Only three daily measurements were performed in patients without fever. In each case with fever measured and especially in cases with antipyretics treatment a much more frequent protocol of temperature measurement was introduced. Temperature measurements were taken orally when patients were fully alert and rectally upon reduced consciousness. In any case of fever detected orally, temperature was taken three times at the ear to assure the measurement accuracy. As the ear site quickly responds to changes in the set point temperature, it is a preferable and recommendable site for measurement of body temperature [29]. As the difference between ear and rectal measurements is of less than 0.2-0.3°C, we did not expect it to change the results of the study [29]. Due to the deleterious effect of fever on patients in the setting of acute ICH, every patient with fever above 38.3°C was treated promptly with a variety of temperature lowering agents. In such a case, temperature measurements were taken an hour later to ensure temperature control. In unconscious patients with fever above 40°C we have used ice water baths and cold saline infusions. In accordance with previous studies on central fever [21,30], presence of fever was defined as any temperature measurement ≥38.3°C. Time elapsed from symptom onset to temperature elevation, peak temperature measurement and time elapsed to peak temperature were collected during the first week of hospitalization. All hyperthermic patients underwent serial chest x-rays, blood counts and repeated cultures of blood, urine, respiratory secretions, stool and shunt fluid if applicable. All patients also had PCR for Clostridium in order to identify infectious causes.

Strict anti-infectious measures are taken on a permanent basis in our neurological ICU. These strict measures include frequent hand wash by the healthcare personnel in general including and during procedures in particular. Intravenous line catheters are replaced on a regular basis according to a standardized internal protocol. In order to avoid microaspirations leading to aspiration pneumonia, every patient who develops dysphagia is inserted with a nasogastric tube without delay. Respiratory machines and equipment are being closely monitored in every patient by a special team. Regarding catheter associated urinary tract infection (CAUTI), duration of catheter insertion and unnecessary repositioning has been minimized as much as possible in order to prevent bacteriuria and potential infection.

For the purposes of the current study the patients were divided into 3 groups: a. Infectious Fever (InF) - defined as any fever measurement ≥ 38.3°C with clinical and a clear laboratory or radiological evidence of infection. Hence, only patients with a positive chest X ray or a positive laboratory as bacterial culture growth or a positive PCR were regarded as InF b. Central Fever (CF) – defined as peak fever measurement ≥ 38.3°C without evidence of infection. c. No Fever (NoF) – defined as no fever measurements ≥ 38.3°C during the first week of admission. Patients with alternative identified causes of noninfectious fever such as drug fever, venous thromboembolism and blood transfusion reactions were excluded. We chose to make the temperature cut at 38.3 as it was chosen by Commichau et al. [21] for the purpose of comparison to our study.

All data included in our stroke registry is used for quality control assurance and functional deficits before admission and at 90 days post infarct were evaluated with the modified Rankin Scale (mRS) score as part of our standard protocol. Favorable outcome was defined as a mRS ≤ 2.

Statistical evaluations were performed with the SPSS PASW 18 package (IBM USA). Data was compared using student’s t-test for continuous variables or chi square tests for categorical variables. mRS data was dichotomized into 0–2 or >3. Regression analysis controlling for variables that yielded a p value of <0.1 on the univariate analysis, was used to determine factors associated with outcome. The Kaplan-Meier Survival analysis with the log-rank test was applied for assessing the effect of a single, categorical variable on survival. Assessment of several variables (categorical & numerical) on survival was carried out using the Cox-Regression model. In this model the Adjusted Hazard Rate (HR) with its 95% Confidence interval was calculated. All statistical tests applied were two-tailed, and p < 0.05 was considered statistically significant.

## Results

Out of 141 patients admitted for ICH during the study period, 95 patients had spontaneous ICH and full data sets and therefore included in the study. Excluded patients had traumatic ICH (8), ischemic stroke with hemorrhagic transformation (16), tumor (4) or incomplete data sets (18).

Of the included 95 patients with full data sets, 39 (41%) had at least one episode of fever (≥38.3°C) during the first week of hospitalization. Of those, 9 had evidence for infection and were classified as InF (9%) while the remaining 30 were classified as CF (32%).

In selected patients (n = 30) CT angiography and CT venography were added to NCCT. When needed, a dedicated angiogram was further performed to rule out vascular malformations (n = 3). Patients with confirmed secondary ICH (e.g. tumors or malformations etc.) were excluded (n = 5) from the study. Furthermore, since the hematoma can obscure underlying structural etiologies and in order to make an accurate diagnosis, a follow up MRI was performed on 16 patients. In one of these patients, in an MRI imaging performed a year after the ICH and after a full absorption of the hematoma, a small underlying Cavernoma was found. This patient was excluded from the statistical analysis.

The presumed etiology of the ICH varied. Hypertension was present in 73 of the patients. Sixteen patients were treated with anticoagulants and 5 of these patients were also taking antiplatelet medications. Of the remaining patients, 39 were treated with a single antiplatelet. Thirty two patients had lobar ICH. In five patients a follow up MRI was performed and in four of them typical radiological features of cerebral amyloid angiopathy were seen.

The baseline characteristics of included patients are presented in Table 1. The median age was 76.5 (range 34–98) and 44% were females. The median hemorrhage volume was 22.1 ml (range 0.5-277). The median admission NIHSS was 9 (range 0–24). The bleeding also involved SAH and IVH components in 16.7% and 47.1% of the patients respectively.

**Table 1:** **Baseline characteristics**

**Variable**	**CF (n = 30)**	**InF (n = 9)**	**NoF (n = 56)**	**P(CF vs. InF)**	**P(CF vs. NoF)**
Age (mean ± SD)	73.67 ± 13.64	75.78 ± 11.45	72.71 ± 12.02	0.676	0.739
Male (%)	40	66.7	57.1	0.159	0.13
Hypertension (%)	75.9	88.9	76.8	0.402	0.924
Beta blockers (%)	21.4	33.3	30.4	0.657	0.387
Ca channel blockers (%)	32.1	33.3	28.6	1	0.736
ACEi/ARB (%)	50	33.3	39.3	0.462	0.35
Diuretics (%)	21.4	11.1	8.9	0.656	0.168
Smoking (%)	20	0	17.3	0.56	0.762
Diabetes Mellitus (%)	29.6	22.2	33.9	1	0.153
Previous CVA (%)	25	11.1	14.3	0.649	0.227
Previous ICH (%)	7.1	11.1	3.6	1	0.598
Antiplatelet treatment (%)	57.1	33.3	50	0.269	0.537
Anti-Coagulation (%)	10.3	22.2	14.3	0.574	0.742
INR (mean ± SD)	1.2 ± 0.583	1.48 ± 0.837	1.41 ± 1.255	0.27	0.404
PTT (mean ± SD)	26.79 ± 5.12	30.55 ± 9.91	29.33 ± 8.09	0.302	0.138
Platelets count (mean ± SD)	232.3 ± 72.58	177.5 ± 31.04	231.57 ± 93.6	0.003	0.971

ACEi- Angiotensin Converting Enzyme inhibitors, ARB- Angiotensin Receptor Blockers.

There were no differences in baseline characteristics between the CF and NoF groups, while the InF and CF groups differed only in that lower platelet counts were noted in the InF group (p = 0.003, Table 1).

The median time from ICH onset to first fever recording was 24 hours (range 0–120) and the median time for fever peak was 48 hours (range 0–150). Correlation analysis showed that fever peak was higher when the fever started earlier (Pearson Correlation ([PC)] = −0.412, p = 0.009) and this showed higher correlation when tested only on CF group (PC = −0.519, p = 0.003).

The time to fever onset and time to peak temperatures were not significantly different between CF and InF patients but peak temperatures were significantly higher in the CF group (p = 0.035).

**Figure 1:**
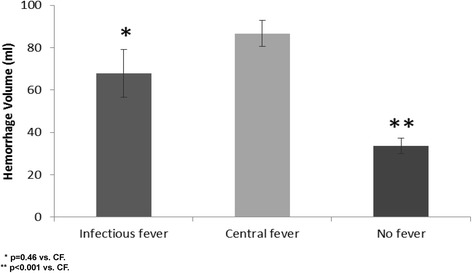
**Mean hemorrhage volumes.** Bar graph showing the mean hemorrhage volumes in patients with infectious (InF), central (CF) and no (NoF) fever groups.

The only factors associated with CF in the univariate analysis (Table 2) were the presence of IVH (70% in CF vs. 30.4% in NoF, p < 0.001) and larger ICH volumes (86.7 ± 66.5 ml in CF vs. 33.7 ± 54.4 ml in NoF, Figure 1 p < 0.001). There was no significant difference between CF and InF in all other ICH attributes and the presence of SAH and hematoma location were not associated with increased chances for developing CF although basal ganglia (BG) and thalamic involvement showed a trend towards significance.

**Table 2:** **ICH characteristics**

**Variable**	**CF (n = 30)**	**InF (n = 9)**	**NoF (n = 56)**	**P (CF vs. InF)**	**P (CF vs. NoF)**
Any lobar involvement (%)	63.3	66.7	44.6	1	0.098
Any BG involvement (%)	76.7	77.8	57.1	1	0.072
Any thalamic involvement (%)	43.3	22.2	23.2	0.437	0.053
Any Cerebellar involvement (%)	3.3	0	10.7	1	0.413
Any BS involvement (%)	6.7	11.1	3.6	0.556	0.608
Volume (mean ± SD) [ml]	86.7 ± 66.5	67.7 ± 67.4	33.7 ± 54.4	0.46	<0.001
IVH (%)	70	55.6	30.4	0.447	<0.001
SAH (%)	23.3	11.1	14.3	0.653	0.292

BG – Basal ganglia, BS- Brainstem, IVH – Intraventricular hemorrhage, SAH – Subarachnoid hemorrhage.

After entering these candidate variables into a stepwise forward logistic regression model, IVH (adjusted OR = 4.667, 95% CI 1.658-13.135) and larger hematoma volumes (adjusted OR = 1.013/ml, 95% CI 1.004-1.021) were identified as significant independent risk factors for developing CF.

**Figure 2:**
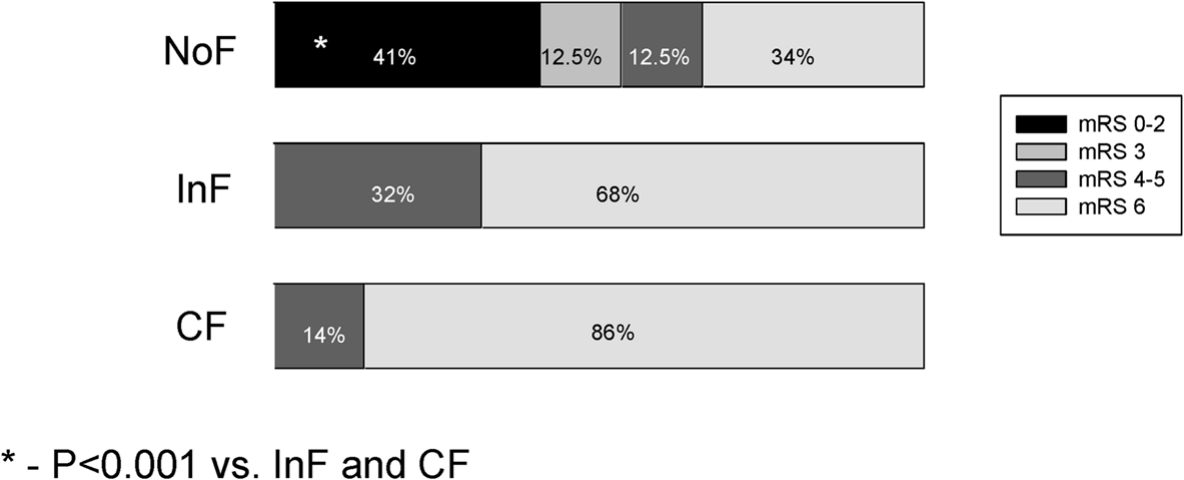
**Outcome at 90 days post ICH.** Bar graphs showing percentage of patients in each modified Rankin Scale category at 90 days after ICH.

Chi-Square analysis of dichotomized mRS data (0–2 versus 3–6) revealed that CF was associated with unfavorable outcome in 100% of cases while NoF was associated with unfavorable outcome in only 46.9% of patients at 90 days post event (Figure 2; p < 0.001).

None of the patients in the InF group had favorable outcomes at day 90 and outcome data of this group did not differ from the CF group (Figure 2). Combining all febrile groups into a single group yielded similar results regarding outcome as those of each individual febrile group.

**Figure 3:**
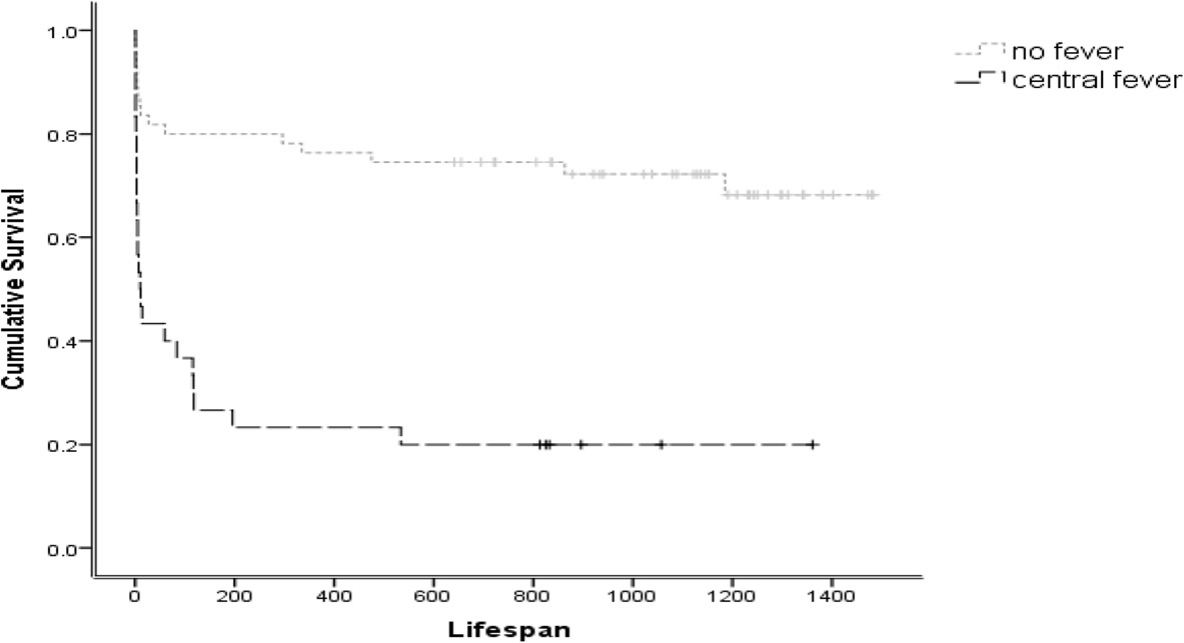
**Survival analysis.** Kaplan Meyer survival curves comparing patients with central (CF) or no (NoF) fever.

The overall mortality in the current cohort was 49%. Increasing temperatures were associated with higher mortality rates (mean fever peak for survivors 38.5°C and 39°C for deceased, p = 0.035). Mortality rates were significantly higher among CF patients compared to those without fever (80% CF vs. 29.1% respectively; p < 0.001). After entering the presence of CF, hematoma volumes and presence of IVH into forward stepwise Cox-Regression analysis, CF (HR = 3.258, 95% CI 1.67-6.37) and ICH volume (HR = 1.01/ml, 95% CI 1.007-1.013) were identified as significant independent risk factors for mortality. The Cox regression model was also used to assess the effect of CF on lifespan. Again, after entering the presence of CF, hematoma volumes and presence of IVH into forward stepwise Cox-Regression analysis for lifespan, CF (HR = 3.021, 95% CI 1.49-6.85) and ICH volume (HR = 1.01/ml, 95% CI 1.005-1.013) were identified as significant independent risk factors for a shorter lifespan. Life span analysis using Kaplan-Meir survival curves (Figure 3) demonstrated significantly higher mortality rates among CF patients compared with NoF (mean projected lifespan: 315 days for CF and 1100 days for NoF, p < 0.001). No significant difference in mortality was found between CF and InF.

## Discussion

The main findings of the current study are that 30% of patients suffering from ICH developed CF [17,21] and that CF is associated with poor outcome and increased mortality rates in these patients.

The current results are in agreement with previous studies that identified the presence of IVH and increasing ICH volume as predictors of CF in patients with SAH and ICH [17,21,26,30,31]. More recently, Rincon et al. [32] described factors associated with fever in patients admitted to neurological ICU and identified hypertension, baseline hematoma volume, intraventricular-hemorrhage, pneumonia, and hematoma growth as markers for development of fever. Another study [25] included 282 patients with ischemic infarcts and 48 patients with ICH. Fever occurred in 37.6% of patients and 14.8% had fever without a documented infection. In multivariate analysis, age, Scandinavian Stroke Scale score and mass effect were found to b significantly associated with fever. In a logistic regression analysis, the only factor predictive of CF versus infection was earlier onset of fever. These findings were recently supported by Hocker et al. [30] who found that CF is more likely to occur within 72 hours of admission to the neurologic ICU. However, the specific impact of CF on outcome in ICH was studied less often and most previous studies lumped ICU patients with fever into a single group.

Previous studies suggest that the association between intraventricular involvement and CF may involve prostaglandin stimulation of the thermoregulatory pathways [22-24] and especially the hypothalamus or direct compression of thermo-sensitive neurons in the brain stem or hypothalamus [27,33]. Interestingly, the current study found no significant correlation between ICH location and occurrence of CF although thalamic and basal ganglia involvement showed a trend towards being significantly more common in patients with CF. This is not surprising when taking into account the proximity of these structures to the third ventricle and the increased incidence of IVH occurrence with such hemorrhage types. Therefore, it appears plausible that future studies including larger numbers of patients may identify hematoma locations such as the thalamus as a potential risk factor for developing CF.

Importantly, peak fever was significantly higher in CF patients compared with InF. In addition, correlation analysis revealed that the earlier the fever started the higher the peak reached. These findings are in agreement with the common notion that CF develops within the first 24 hours from brain injury and reaches high peaks of 40-42°C [20,25]. However, it also shows that a definition based on the mentioned timeline is difficult to establish, probably due to the spectrum of structural damage that might lead to different levels of irritation in the thermoregulatory centers as well as to the presence of confounding variables such as infections or drug reactions.

Importantly, there were no differences in mortality and unfavorable outcome rates between the CF and InF groups and both groups fared worse than afebrile patients. This may suggest that it is the actual presence of fever that determines prognosis and not the actual cause of the increased temperature. Indeed, hyperthermia is associated with poor outcome in many forms of experimental and clinical brain injury [11,13,14,16,18,19]. The mechanisms responsible for the poor outcome in hyperthermic patients remain unknown but may include increased intracranial pressure [19], reduced cerebral blood flow [19] and increase in inflammatory cytokines and axonal death [12].

Our study has several potential limitations. First, the numbers of included patients with InF was surprisingly small and may have hampered full analysis of differences between the InF and CF groups [34]. However, we believe that the paucity of infections detected in our study stems from adopting strict anti-infectious strategies in our intensive care unit as well as in our Neurology department since similar and even lower rates of infection were observed for patients with ischemic stroke. Second, this was a retrospective analysis of the data rather than a prospective randomized study and this may have introduced a bias. However, the data was accrued prospectively which may limit the bias effect. Third, we did not evaluate potential treatments for CF or InF and treatment effects on outcome. We believe that given the relatively high frequency and the large observed impact of CF on outcome, these parameters should be the subject of future randomized studies.

While previous studies have either focused on ICU patients in general or on stroke patients combining both ischemia, ICH, subdural and Subarachnoid hemorrhage (SAH), our study specifically focused on patients with primary spontaneous ICH. Our results are in agreement with previous studies in finding the presence of IVH and increasing ICH volume as predictors of CF in patients with SAH and ICH.

Previously it was believed that CF develops within the first 24 hours from brain injury and reaches a peak of 40-42°C [20,25]. Our findings are in partial agreement with these findings. In regard to temperature, our study showed that peak fever was significantly higher in CF patients compared with InF and correlation analysis revealed that the earlier the fever started the higher the peak reached. In regard to the timeframe of CF, our findings were much like those of Hocker et al. who found that CF is more likely to occur within 72 hours of admission to the neurologic ICU but can also occur later on. We presume that a definition based on the mentioned timeline is difficult to establish, probably due to the spectrum of structural damage that might lead to different levels of irritation in the thermoregulatory centers.

## Conclusion

In conclusion, CF is common after spontaneous ICH and the risk of developing CF is increased in patients with larger ICH and in those with IVH extension. CF is associated with increased mortality rates, shorter lifespan and with reduced chances for favorable outcome and should be considered as a poor prognostic factor in patients with ICH. Future studies are needed to evaluate treatments for CF in an effort to minimize ICH induced damage and poor outcome.

## Abbreviations

SAH: Subarachnoid hemorrhage
TBI: Traumatic brain injury
IVH: Intraventricular hemorrhage
CF: Central fever
InF: Infectious fever
